# Choroidal thickness and granulocyte colony-stimulating factor in tears improve the prediction model for coronary artery disease

**DOI:** 10.1186/s12933-022-01538-0

**Published:** 2022-06-09

**Authors:** José Lorenzo Romero-Trevejo, Lourdes Fernández-Romero, Josué Delgado, Erika Muñoz-García, Andrés Sánchez-Pérez, Mora Murri, Mario Gutiérrez-Bedmar, Manuel Francisco Jiménez-Navarro

**Affiliations:** 1 Department of Ophthalmology, Virgen de la Victoria University Hospital, Campus de Teatinos, s/n. 29010 Malaga, Spain; 2 Department of Medicine and Dermatology. School of Medicine, University of Malaga, Campus de Teatinos, s/n. 29010 Malaga, Spain; 3 Malaga Biomedical Research Institute-IBIMA, Virgen de La Victoria University Hospital, Campus de Teatinos, s/n. 29010 Malaga, Spain; 4 Department of Heart and Cardiovascular Pathology, Virgen de La Victoria University Hospital, Campus de Teatinos, s/n. 29010 Malaga, Spain; 5 CIBERCV Cardiovascular Diseases, Carlos III Health Institute, Madrid, Spain; 6 CIBEROBN Obesity and Nutrition, Carlos III Health Institute, Madrid, Spain; 7 Department of Preventive Medicine and Public Health. School of Medicine, University of Malaga, Campus de Teatinos, s/n. 29010 Malaga, Spain

**Keywords:** Coronary artery disease, Choroidal thickness, Granulocyte colony-stimulating factor, Cardiovascular prevention, ROC curves, Predictive model

## Abstract

**Background:**

Coronary artery disease (CAD) detection in asymptomatic patients still remains controversial. The aim of our study was to evaluate the usefulness of ophthalmologic findings as predictors of the presence of CAD when added to cardiovascular classic risk factors (CRF) in patients with acute coronary cardiopathy suspicion.

**Methods:**

After clinical stabilization, 96 patients with acute coronary cardiopathy suspicion were selected and divided in two groups: 69 patients with coronary lesions and 27 patients without coronary lesions. Their 192 eyes were subjected to a complete routine ophthalmologic examination. Samples of tear fluid were also collected to be used in the detection of cytokines and inflammatory mediators. Logistic regression models, receiver operating characteristic curves and their area under the curve (AUC) were analysed.

**Results:**

Suggestive predictors were choroidal thickness (CT) (OR: 1.02, 95% CI 1.01–1.03) and tear granulocyte colony-stimulating factor (G-CSF) (OR: 0.97, 95% CI 0.95–0.99). We obtained an AUC of 0.9646 (95% CI 0.928–0.999) when CT and tear G-CSF were added as independent variables to the logistic regression model with cardiovascular CRF: sex, age, diabetes, high blood pressure, hypercholesterolemia, smoking habit and obesity. This AUC was significantly higher (p = 0.003) than the prediction derived from the same logistic regression model without CT and tear G-CSF (AUC = 0.828, 95% CI 0.729–0.927).

**Conclusions:**

CT and tear G-CSF improved the predictive model for CAD when added to cardiovascular CRF in our sample of symptomatic patients. Subsequent studies are needed for validation of these findings in asymptomatic patients.

## Background

To date, there have been no in-depth studies into the relationship between ophthalmological findings and coronary artery disease (CAD), which may seem paradoxical considering the anatomical and physiopathological characteristics shared by both systems [1, 2]. Cardiovascular diseases (CVD) are the leading cause of death worldwide, and accounted for almost 18 million deaths according to estimations made in 2017 [3]. However, the global burden of CVD is not just a health issue; for health systems, it represents a financial challenge that is expected to undergo exponential growth in the future [4]. For these reasons, the availability of predictive tools that would help reduce the prevalence and incidence rates of CVD are a key element to improve both patient health and the sustainability of healthcare services, bearing in mind the large number of people who would benefit no matter how small the preventive measure to apply.

Since the Framingham Heart Study identified the classic risk factors (CRF) for developing CVD [5], numerous cardiovascular risk prediction models have received validation and gained popularity. These models consider factors such as the presence of high blood pressure, hypercholesterolemia, diabetes, obesity, smoking habit, alcohol consumption, diet and physical exercise among others [6]. However, the prediction models available to date have manifest limitations, chiefly because their correct application among all populations worldwide is not possible, and it would be ideal if each community could develop its own model [7], or because they have difficulty detecting the risk, for instance, among women. For these reasons, screening to detect asymptomatic CAD still remains controversial, and updated guidelines continue to emphasise the need for its early detection in order to establish prognostic and therapeutic strategies designed to reduce morbidity and mortality among such patients [8].

Its particular structure and the ease of access to the eye make this organ a candidate for obtaining potentially useful parameters in this sense, bearing in mind its involvement in a multitude of systemic processes whose first sign may even show itself as an ophthalmological symptom [9]. Moreover, changes in the cardiovascular system have also been related with signs that are visible in the eye, making this organ a window that provides quick access to the cardiovascular system thanks to the ease with which it is possible to see findings [10]. Finally, considering that some CRF for CVD such as diabetes, high blood pressure or hyperlipidaemia, produce typical, specific lesions in the eye fundus, there is a clearly established relationship between this organ and cardiovascular conditions. Nevertheless, to date, research has shown no clear relationship between CVD and the presence of abnormalities in other ocular structures or the expression of different inflammatory mediators in tears.

The objective of this work was, therefore, to analyse the utility of the ophthalmological findings and biomarkers in tears as predictors for the detection of CAD. This would improve on existing models, enabling such predictors to become a useful tool in habitual clinical practice since they represent an unexpensive and less invasive way to categorize and efficiently manage these patients, not only from the perspective of patient health but also of healthcare resource management.

## Methods

### Study population

Patients of this observational, case–control, single centre study were assessed in the Cardiology and Ophthalmology Departments of the Virgen de la Victoria University Hospital of Malaga between the months of January-March 2019. Inclusion criteria for the study were suspicion of having a coronary heart disease based on presentation of oppressive chest pain at rest or on with an increase in activity or stress, with or without the presence of dyspnoea at the time of the consultation. Exclusion criteria were the existence of advanced kidney failure (defined as a glomerular filtration rate lower than 30 ml/min), suffering from any disease that reduced life expectancy to less than one year, having presented another cardiovascular event before this study, suffering from any type of retinal disease (including diabetic or hypertensive retinopathy, diabetic macular oedema, vein occlusions, retinal dystrophies, epiretinal membranes, vitreomacular traction, age-related macular degeneration or central serous corioretinopathy), the presence of amblyopia in either eye or having undergone retinal photocoagulation treatment in the past.

### Cardiovascular examination

To confirm the existence of CAD, the selected patients underwent coronary angiogram and/or a computerised tomography scan of the coronary arteries. Depending on the angiogram results, we defined CAD as such with findings of a minimum involvement of 70% in any major epicardial artery (right coronary, anterior descending or marginal circumflex) or more than 50% in the left main coronary artery. Based on the computerised tomography of coronary arteries, we defined CAD as such where there was involvement of at least 50% of any coronary artery. After these results, patients we distributed into two study subject groups: patients without coronary lesions and patients with coronary lesions. After their clinical stabilisation, patients underwent a full ophthalmological examination within the first ten days after the onset of the clinical symptoms that were the reason for the cardiovascular study.

### Ophthalmological examination

The same ophthalmologist conducted the ophthalmological examinations at the same time of day (15–18 pm). Before carrying out examinations, we collected data about the CRF for the onset of cardiovascular disease for each patient, such as sex, age, presence of diabetes, high blood pressure, hypercholesterolemia, smoking habit or obesity. The ophthalmological examination consisted of evaluating different clinical parameters, such as best-corrected visual acuity (BCVA) with numerical eye charts, intraocular pressure (IOP) using a Perkins Mk3 tonometer (Haag-Streit, Essex, UK), Schirmer's test (ST) using Schirmer-Plus^®^ strips (GECIS, Neung-sur-Beuvron, France), central corneal thickness (TCCT and PCCT), axial length (AL) and several variables obtained by means of optical coherence tomography (OCT) and OCT-angiography. In addition, as laboratory variables, we studied the presence of cytokines and other inflammatory mediators in samples of tears.

Central corneal thickness was automatically measured with the Orbscan^®^ IIz (Bausch and Lomb, Rochester, USA) topographer and, after administration of tetracaine and oxybuprocaine anaesthetic eye drops, manually with the OcuScan^®^ RxP (Alcon Laboratories, Texas, USA) ultrasound pachymeter, with calculation of the arithmetic mean of 10 consecutive measurements taken from each eye with the patient seated. AL was automatically obtained with partial coherence interferometry using the IOLMaster^®^ 500 (Carl Zeiss Meditec AG, Jena, Germany) optical biometer. Cirrus™ (Carl Zeiss Meditec AG, Jena, Germany) high-definition (HD)-OCT was used for OCT, with the equipment providing automatically obtained values for central macular thickness (CMT), macular cube volume (MCV), mean macular thickness (MMT), retinal nerve fibre layer thickness (RNFLT), ganglion cell layer thickness (GCLT) and ganglion cell layer minimum thickness (GCLMT). Choroidal thickness (CT) was calculated manually using the device’s ruler from the images it produced, and was defined as the distance between the external hyper-reflective band of the retinal pigment epithelium and the internal hyper-reflective band of the sclera. This was expressed as the arithmetic mean of 10 measurements: 5 on the horizontal axis and 5 on the vertical axis, with a separation of 500 micra between them to either side of subfoveal area. For inclusion in this study, the minimum acceptable image quality was 6 out of 10. The OCT-angiography was performed with DRI OCT Triton™ plus (Topcon Medical Systems, Oakland, USA) and the device software automatically provided the values for central vascular density in the different layers of the retina (ILM, RNFL-GCL, GCL-IPL, IPL-INL). In the case of prior corneal surgery or a clinically significant eye cataract, we excluded the values obtained for these eyes for BCVA, IOP, TCCT, PCCT and the OCT and OCT-angiography.

Conducting ST served also to collect tear samples from each patient. The paper strip was placed to rest in the lower base of each eye, without previous application of topical anaesthetic, and left for 5 min. The study excluded samples from any eyes where moisture on the strip measured less than 6 mm after this period. Valid samples were immediately frozen at -80º C after collection, and remained at that temperature until analysis. For protein elution each paper sample was cut into small pieces that were introduced into 100 μl of PBS with Tween^®^ 20 at 0.3%, bovine albumin serum at 0.5% and protease inhibitor, for overnight incubation 4º C before collection of supernatant. Measurement of the total amount of protein in each sample used the NanoDrop™ One (ThermoFischer Scientific, Waltham, MA, USA) spectrophotometer with absorbance measured at 280 nm. Detection of cytokines and inflammatory mediators followed the instructions of the Bio-Plex Pro ™ kit Human Cytokine 27-Plex Assay (Bio-Rad Laboratories, Hercules, CA, USA). The 27 cytokines and inflammatory mediators analysed were interleukin (IL)-1β, IL-1RA, IL-2, IL-4, IL-5, IL-6, IL-7, IL-8, IL-9, IL-10, IL-12p70, IL-13, IL-15, IL-17, basic fibroblast growth factor (FGF), eotaxin (EO), granulocyte colony-stimulating factor (G-CSF), granulocyte–macrophage colony-stimulating factor (GM-CSF), interferon (IFN)-γ, chemokine (IP)-10, monocyte chemoattractant protein (MCP)-1, macrophage inflammatory protein (MIP)-1α, platelet-derived growth factor (PDGF)-bb, MIP-1β, chemoquine ligand 5 (RANTES), tumour necrosis factor (TNF)-α and vascular endothelial growth factor (VEGF).

### Statistical analysis

Data were analysed with Stata^®^ 17 (StataCorp LLC, Texas, USA). Characteristics of participants were described as a mean and standard deviation for continuous variables and percentage for categorical variables. Group comparisons were carried out using a Student’s t-test or Chi-squared test as appropriate.

For each variable associated with the presence of coronary lesions in the bivariate analysis, a logistic regression model was estimated that included the CRF: sex, age, diabetes, high blood pressure, hypercholesterolemia, smoking habit and obesity. We assessed the statistical significance of each variable using the likelihood-ratio test based on the models with and without the variable (only with CRF). For variables that were statistically significant, we estimated the area under curve (AUC) associated with the logistic regression model. To estimate the predictive capacity of these variables, we compared the areas under curve using the Chi-squared test, taking the AUC of the logistic regression model that only included CRF. Finally, a logistic regression prediction model was constructed that included the CRF and all variables whose logistic regression models showed an AUC higher than the only-CRF model.

## Results

A total of 96 patients were recruited and distributed into the two study subject groups. There were 27 patients without coronary lesions and 69 patients with coronary lesions. The total 192 eyes examined were individually analysed. Table 1 shows the distribution of absolute and relative frequency of the number of eyes included in each group by CRF studied.

**Table 1:** Characteristics of participants according to the presence of coronary lesions

	All	Without coronary lesions	With coronary lesions	p (Chi-squared test)
Number of eyes	192	54	138	
Female sex (%)	54/192 (28)	**32/54 (59)**	**22/138 (16)**	** < 0.001**
Age in years (mean ± SD)	58.69 ± 9.49	57.33 ± 10.67	59.22 ± 8.93	0.22^a^
Diabetes (%)	68/192 (35)	18/54 (33)	50/138 (36)	0.71
High blood pressure (%)	84/192 (44)	20/54 (37)	64/138 (46)	0.24
Hypercholesterolemia (%)	70/192 (36)	24/54 (44)	46/138 (33)	0.15
Smoking habit (%)	78/192 (41)	**12/54 (22)**	**66/138 (48)**	**0.001**
Obesity (%)	78/192 (41)	24/54 (44)	54/138 (39)	0.50

*SD* standard deviation. Statistically significant results are shown in bold (p<0.05).

^a^Student’s *t*-test

Table 2 provides the results of the comparative study between the two groups of patients in terms of the ophthalmological variables assessed. We excluded the cytokines IL-2, IL-10, IL-12p70, IL-15, FGF and VEGF before the study because the laboratory tests found no detectable values of them. After the comparative study, the variables finally chosen to carry out the prediction model because they showed statistically significant differences between the mean values for both groups were axial length, CT, IL-8, G-CSF, MCP-1, MIP-1α and RANTES.

**Table 2:** Comparative study for the measured clinical and laboratory ophthalmological variables

Variable	Without coronary lesions	n	With coronary lesions	n	p (Student’s *t*-test)
BCVA	0.95 ± 0.13	54	0.97 ± 0.07	134	0.36
IOP	15.13 ± 3.93	54	14.88 ± 3.11	136	0.64
ST	16.24 ± 10.28	54	15.20 ± 9.87	138	0.52
TCCT	546.28 ± 32.61	50	553.16 ± 37.76	126	0.26
PCCT	543.08 ± 32.95	52	549.79 ± 36.54	130	0.25
AL	**23.35 ± 1.48**	**54**	**23.77 ± 1.16**	**120**	**0.04**
CMT	261.24 ± 22.77	54	267.33 ± 26.10	132	0.14
MCV	10.14 ± 0.39	54	10.14 ± 0.56	132	0.97
MMT	281.54 ± 10.83	54	281.44 ± 15.46	132	0.97
RNFLT	89.54 ± 9.72	54	89.31 ± 12.39	131	0.90
GCLT	81.20 ± 8.44	54	79.73 ± 10.84	133	0.37
GCLMT	76.63 ± 12.19	54	74.14 ± 17.36	133	0.34
CT	**260.74 ± 44.00**	**54**	**308.29 ± 69.92**	**131**	** < 0.001**
ILM	20.68 ± 4.29	41	20.70 ± 5.40	52	0.99
RNFL-GCL	21.27 ± 4.32	41	21.45 ± 5.69	52	0.87
GCL-IPL	26.08 ± 4.71	41	25.86 ± 5.73	52	0.85
IPL-INL	32.64 ± 5.73	41	30.98 ± 5.52	52	0.16
IL-1β	0.38 ± 0.19	39	0.45 ± 0.81	92	0.60
IL-1RA	4723.71 ± 2361.33	34	4539.61 ± 2158.62	95	0.68
IL-4	0.37 ± 0.21	44	0.35 ± 0.16	102	0.45
IL-5	4.60 ± 2.75	12	3.66 ± 1.50	30	0.16
IL-6	2.54 ± 3.34	43	2.29 ± 4.00	102	0.73
IL-7	4.85 ± 2.03	45	5.01 ± 2.26	106	0.69
IL-8	**112.42 ± 141.78**	**45**	**66.68 ± 87.25**	**107**	**0.02**
IL-9	4.57 ± 3.38	29	4.84 ± 3.06	59	0.70
IL-13	0.43 ± 0.25	25	0.40 ± 0.21	75	0.60
IL-17	2.37 ± 0.97	2	2.10 ± 1.80	14	0.85
EO	0.62 ± 0.23	45	0.58 ± 0.22	107	0.24
G-CSF	**44.38 ± 53.10**	**25**	**20.98 ± 29.01**	**59**	**0.01**
GM-CSF	0.58 ± 0.28	28	0.62 ± 0.28	75	0.57
IFN-γ	65.86 ± 23.69	45	58.62 ± 24.41	107	0.09
IP-10	2062.35 ± 1174.51	42	2338.23 ± 1109.79	100	0.19
MCP-1	**21.67 ± 20.85**	**45**	**13.72 ± 16.48**	**107**	**0.01**
MIP-1α	**0.74 ± 1.51**	**45**	**0.39 ± 0.61**	**106**	**0.05**
PDGF-BB	19.49 ± 11.51	18	21.16 ± 11.49	35	0.62
MIP-1β	4.06 ± 3.92	42	4.25 ± 6.68	97	0.87
RANTES	**22.36 ± 15.69**	**45**	**33.85 ± 27.22**	**107**	**0.009**
TNF-α	13.26 ± 11.69	13	13.38 ± 11.38	25	0.98

Data are shown as mean ± standard deviation. Statistically significant results are shown in bold (p<0.05).

*n* Number of eyes; *BCVA* Best corrected visual acuity; *IOP* Intraocular pressure; *ST* Schirmer’s test; *TCCT* Topography central corneal thickness; *PCCT* Pachymetry central corneal thickness; *AL* Axial length; *CMT* Central macular thickness; *MCV* Macular cube volume; *MMT* Mean macular thickness; *RNFLT* Retinal nerve fibre layer thickness; *GCLT* Ganglion cell layer thickness; *GCLMT* Ganglion cell layer minimun thickness; *CT* Choroidal thickness; *ILM* Central vascular density under the inner limiting membrane; *RNFL-GCL* Central vascular density between retinal nerve fibre layer and ganglion cell layer; *GCL-IPL* Central vascular density between ganglion cell layer and inner plexiform layer; *IPL-INL* Central vascular density between inner plexiform layer and inner nuclear layer; *IL* Interleukin; *EO* Eotaxin; *G-CSF* Granulocyte colony-stimulating factor; *GM-CSF* Granulocyte–macrophage colony-stimulating factor; *IFN* Interferon; *IP* Chemokine; *MCP* Monocyte chemoattractant protein; *MIP* Macrophage inflammatory protein; *PDGF* Platelet-derived growth factor; *RANTES* Chemokine ligand 5; *TNF* Tumour necrosis factor

The logistic regression study found a direct relationship between CT and RANTES and the presence of coronary lesions, and an inverse relationship between IL-8, G-CSF, MCP-1 and the presence of such lesions. Conversely, the ophthalmological variables that, on an individual basis, significantly improved results of AUC when added to the CRF were CT and G-CSF. Table 3 shows the results of this analysis.

**Table 3:** Logistic regression and comparative study between area under curve (AUC) of the prediction models with the classic risk factors (CRF) including, or not, the selected ophthalmological variables

Variable	OR^a^ (CI 95%)	AUC^c^ (CI 95%)	AUC^d^ (CI 95%)
AL	1.13 (0.81–1.58)	0.83 (0.76–0.90)	0.83 (0.76–0.90)
CT	**1.02 (1.01–1.03)**^b^	**0.89 (0.84–0.94)**^e^	**0.83 (0.77–0.90)**
IL-8	**0.99 (0.99–1.00)**^b^	0.86 (0.80–0.92)	0.84 (0.78–0.91)
G-CSF	**0.97 (0.95–0.99)**^b^	**0.91 (0.84–0.97)**^e^	**0.82 (0.72–0.92)**
MCP-1	**0.96 (0.94–0.99)**^b^	0.87 (0.80–0.93)	0.84 (0.78–0.91)
MIP-1β	0.99 (0.91–1.08)	0.87 (0.80–0.93)	0.85 (0.79–0.92)
RANTES	**1.02 (1.00–1.05)**^b^	0.87 (0.81–0.93)	0.84 (0.78–0.91)

*Statistically significant results are shown in bold (p<0.05). OR* Odds ratio; *CI* Confidence interval; *AL* Axial length; *CT* Choroidal thickness; *IL* Interleukin; *G-CSF* Granulocyte colony-stimulating factor; *MCP* Monocyte chemoattractant protein; *MIP* Macrophage inflammatory protein; *RANTES* Chemokine ligand 5

^a^Adjusted by variable and CRF: sex, age, diabetes, high blood pressure, hypercholesterolemia, smoking habit, and obesity

^b^p < 0.05 (likelihood ratio test)

^c^AUC from model adjusted by variable and CRF

^d^AUC from model adjusted only by CRF

^e^Statistically significantly (p < 0.05) higher than AUC from model adjusted only by CRF

**Fig. 1:**
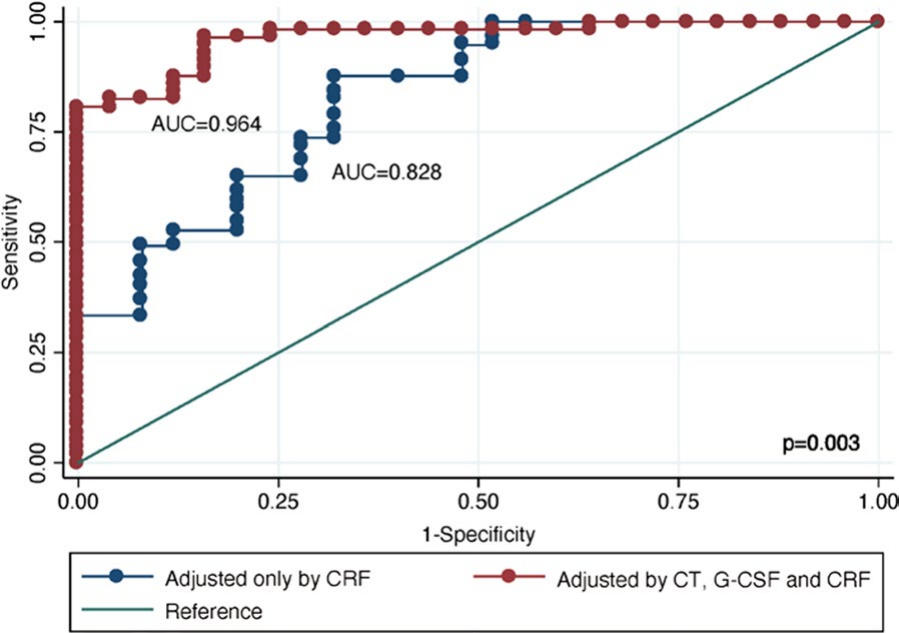
Comparison of receiver operating characteristic (ROC) curves between logistic regression models. AUC: Area under curve. CRF: Classic risk factors. CT: Choroidal thickness, G-CSF: Granulocyte colony-stimulating factor, p-Value: Chi-square test

In the multivariate analysis that included CT and G-CSF along with the other CRF, the AUC obtained was 0.964, which constituted a statistically significant difference in comparison with the AUC value that only considered AUC (Fig. 1, Table 4). The probability of the existence of coronary lesions according to the best model obtained from the studied sample was possible to calculate using the equation ln (Odds of lesion) = − 18.56 + 0.032 × CT—0.067 × G-CSF–3.788 × sex + 0.1923 × age + 2.372 × diabetes + 1.696 × high blood pressure − 2.425 × hypercholesterolemia + 7.357 × smoking habit − 1.209 × obesity. The receiver operating characteristic (ROC) curve analysis revealed that a p ≥ 0.45 would predict the existence of coronary lesions with a sensitivity of 96.49% and a specificity of 84%, allowing the correct classification of 92.68% of patients.

**Table 4:** Area under curve (AUC) for the prediction models with the classic risk factors (CRF) including, or not, the selected ophthalmological variables

Variables	AUC^a^ (CI 95%)	AUC^b^ (CI 95%)	p (Chi-square test)
CT + G-CSF	**0.964 (0.928–0.999)**	**0.828 (0.729–0.927)**	**0.003**

*Statistically significant results are shown in bold (p<0.05). CI* Confidence interval; *CT* Choroidal thickness, *G-CSF* Granulocyte colony-stimulating factor

^a^AUC from model adjusted by CT, G-CSF and CRF: sex, age, diabetes, high blood pressure, hypercholesterolemia, smoking habit, and obesity

^b^AUC from model adjusted only by CRF

## Discussion

The results of our study demonstrate that, among the patients studied, the values for CT and G-CSF in tears improve the AUC of the prediction model for CAD when added to CRF for this disease. These variables have a direct and inverse relationship respectively with the presence of coronary lesions. To the best of our knowledge, this is the first study into the use of two ophthalmological variables, one clinical and the other laboratory-based, to facilitate the detection of CAD. Its added advantage is that obtaining the data is relatively quick, simple and non-invasive in the usual clinical practice.

The choroid consists of a dense network of blood vessels, located between the retina and the sclera, whose function is to supply oxygen and nutrients to the external layers of the retina and pigment epithelium, the subfoveal avascular zone and the optic nerve. In recent years, a growing body of evidence has shown that changes in choroidal microvasculature might be indicative of other systemic diseases that affect blood vessels [11, 12]. Thus, the relationship between the choroid and CVD represents an area of clinical interest and a potential prognostic factor or biomarker for such diseases [1]. Although there is growing body of evidence to support the theory that changes in CT may be of prognostic use, controversy still exists in this regard [13]. Recent studies have suggested that in CAD there is a probable relationship between a decrease in capillary density and choroid blood flow, as well as with a general thinning of choroid [2, 14–17]. Such reports contradict the findings of our study, which indicate that an increase in choroidal thickness is associated with the presence of coronary lesions. However, there are some important aspects of the above-mentioned studies that may justify the disparity. First, in addition to the fact that the number of patients studied was slightly lower than in our research, the inclusion criteria for two of the studies was the presence of coronary slow-flow phenomenon [14, 15], the definition of which excludes the presence of evident obstructive disease and which, therefore, would preclude comparison of the results obtained. Secondly, another of the studies associated the presence of CAD with a decrease of capillary density and choroid flow in general, without referring to thickness [16]. Furthermore, this study shows an unexpectedly more intense vascular density of the external retina among CAD patients, with no determination of whether this increased density could be related with CT or not, an association that still remains uncertain [18]. Thirdly, a most similar study only compares the means between groups, does not adjust the results based on other classical risk factors, and reports an average of the duration of CAD of 23 months [17]. Finally, the work that bears most comparison with ours has shown a decrease in choroidal thickness among patients with CAD. Although the normal spatial pattern of the choroid was maintained, a significantly lower number of patients were used and the study was performed with patients recruited from primary healthcare and ambulatory clinic outpatients [2]. As with the previous report, this means that factors such as the possible effect of a time-lapse between having passed the acute phase of the disease, or daily fluctuations in CT may have had a significant influence on obtaining the data. Our patients, however, were evaluated within the first ten days after the onset of the clinical symptoms of CAD. On the other hand, no significant differences in the subfoveal choroidal thickness among controls and patients with one, two or triple vessel coronary artery disease have also been published [19]. In any case, it is practically impossible to establish the isolated connection between CAD, CT and clinical risk, as a great number of physiological variations, lifestyle, pharmacological treatment and comorbidities such as high blood pressure, dyslipidaemia, diabetes or other inflammatory and autoimmune conditions have been individually associated with the increase or decrease of clinical risk [1, 10]. Given this circumstance, from a clinical perspective, it would be more practical to create and validate prediction models that integrate all of these conditions, as we have undertaken in this work.

G-CSF is a hematopoietic growth factor produced by monocytes, macrophages, fibroblasts, endothelial cells, vascular smooth muscle cells or bone marrow stromal cells that have the capacity to regulate proliferation, differentiation, survival and growth of neutrophil progenitor cells, contributing to the conversion of granulocyte colony-forming units to polymorphonuclear leukocytes, with their subsequent release from bone marrow into the bloodstream [20]. In addition, G-CSF mobilises stem cells from bone marrow to peripheral blood, and induces their differentiation to cardiomyocytes or endothelial cells when they come into contact with damaged areas of the myocardium [21]. Thus, in experimental models of acute myocardial infarction, the animals treated with G-CSF showed a significant increase in survival compared to the ones who did not receive it [22], although other published studies report contradictory findings [23]. Despite the currently existing controversies, having detected that high plasma levels of endogenous G-CSF may predict cardiovascular events regardless of the established risk factors [24], the use of G-CSF as an element that mobilises stem cells to repair myocardial damage, in the treatment of congestive heart failure, or as a mediator in atherosclerosis, is an attractive concept for future research. Such research should particularly focus on the search for feasibility and an acceptable cost–benefit relationship [21, 25, 26]. To the best of our knowledge, this is the first time that research has associated levels of G-CSF in tears with the presence of CAD, showing that an increase in its levels may constitute a protective factor, whereas lower levels are associated with the existence of a possible onset of coronary lesions. These data concur with the results of these previously published studies that report G-CSF as playing an important role in the cardiac regeneration processes after ischaemic damage, although future tests are necessary to determine with greater clarity which mechanisms are involved in these processes.

The prevention of CVD requires the identification of people who are at high risk, in order to propose suitable pharmacological, dietary or lifestyle interventions. Recent decades have seen the development of a multitude of prediction models for the general population, to name but a few: the Framingham [27], SCORE [28], QRISK [29] or, in our country, IberScore [30]. Nevertheless, in most cases their use remains unclear, due to defects in method, incomplete presentation, lack of external validation or heterogeneity in the chosen predictors, or in the definition of the events evaluated [31]. For this reason, in the age of excessive information, future work should focus on the external validation and comparison of existing models, their combination and adaptation for application among local populations, and their extension by means of adding new predictors [31, 32]. To this end, our results propose two new ophthalmological variables: CT and G-CSF in tears. Used in conjunction with the CRF, they offer a prediction model that allows the correct classification of a higher number of patients based on the presence or absence of coronary lesions. Moreover, and thanks to this, transdisciplinary management of these patients would be possible since, in the current context, individuals with CVD do not receive suitable ophthalmological follow-up [1], and unidisciplinary approach may not be optimal when disease causation is complex and health decisions are pressing [33].

The main strength of our study resides in the degree of precision in measuring the ophthalmological variables employed. Among its limitations, the foremost are the use of a homogeneous group of patients who come from a single hospital, the relatively small sample size and the fact that it only studied symptomatic individuals. For all of these reasons, future prospective studies with a higher number of patients are necessary to verify these promising results and evaluate the validity of their application in asymptomatic individuals.

## Conclusions

CT and tear G-CSF improved the predictive model for CAD when added to cardiovascular CRF in our sample of symptomatic patients. Although subsequent studies are needed for validation of these findings in asymptomatic patients, this is the first study into the use of two ophthalmological variables to facilitate the detection of CAD.

## Data Availability

Not applicable.

## Abbreviations

AL: Axial length
AUC: Area under the curve
BCVA: Best corrected visual acuity
CAD: Coronary artery disease
CI: Confidence interval
CMT: Central macular thickness
CRF: Classic risk factors
CT: Choroidal thickness
CVD: Cardiovascular diseases
G-CSF: Granulocyte colony-stimulating factor
GCL: Ganglion cell layer
GCLMT: Ganglion cell layer minimum thickness
GCLT: Ganglion cell layer thickness
HD: High definition
IL: Interleukin
ILM: Inner limiting membrane
INL: Inner nuclear layer
IOP: Intraocular pressure
IP: Chemokine
IPL: Inner plexiform layer
MCP: Monocyte chemoattractant protein
MCV: Macular cube volume
MIP: Macrophage inflammatory protein
MMT: Mean macular thickness
OCT: Optic coherence tomography
OR: Odds ratio
PCCT: Pachymetry central corneal thickness
PDGF: Platelet-derived growth factor
RANTES: Chemokine ligand 5
RNFL: Retinal nerve fibre layer
RNFLT: Retinal nerve fibre layer thickness
ROC: Receiver operating characteristic
SD: Standard deviation
ST: Schirmer’s test
TCCT: Topography central corneal thickness
TNF: Tumour necrosis factor
VEGF: Vascular endothelial growth factor
